# Supplementary material for: Starve a cold or feed a fever? Identifying cellular metabolic changes following infection and exposure to SARS-CoV-2
Source: bioRxiv. 2024 May 23:2024.05.22.595410. Preprint. [Version 1] doi: 10.1101/2024.05.22.595410 (PMC11142155; doi:10.1101/2024.05.22.595410)
Supplement: Supplement 2 [file NIHPP2024.05.22.595410v1-supplement-2.pdf]

bioRxiv preprint doi: https://doi.org/10.1101/2024.05.22.595410; this version posted May 23, 2024. The copyright holder for this preprint (which was not certified by peer review) is the author/funder, who has granted bioRxiv a license to display the preprint in perpetuity. It is made available under aCC-BY-NC-ND 4.0 International license.

Supplementary Figures

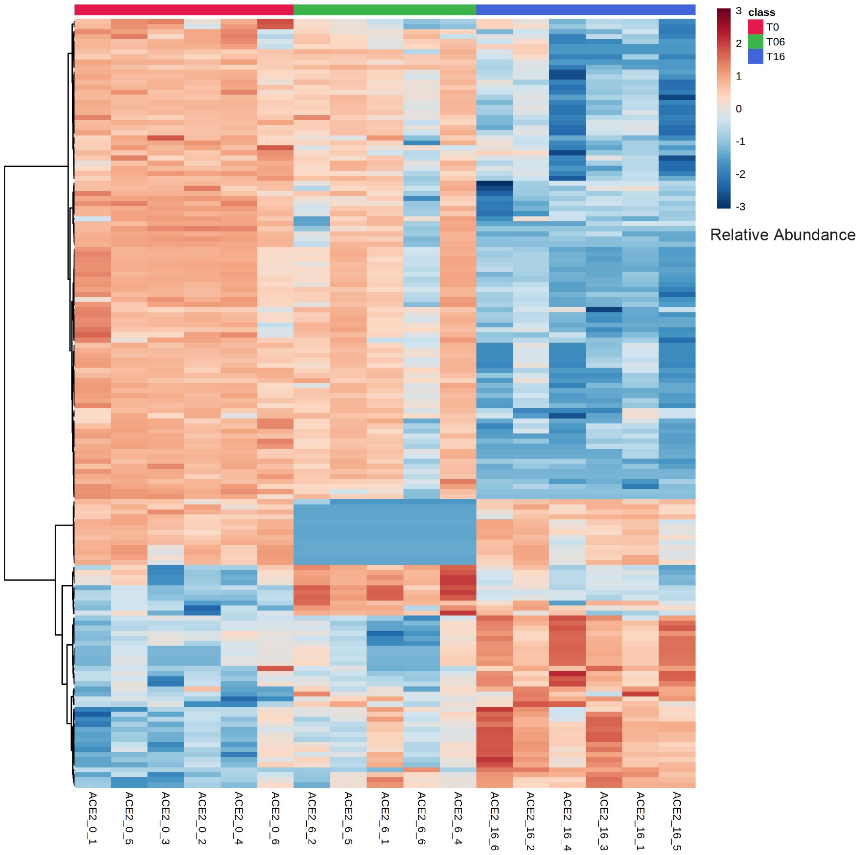

Figure S1: Heatmap analysis of significant metabolites (n = 152) for all ACE2-A549 samples at each time point.

bioRxiv preprint doi: https://doi.org/10.1101/2024.05.22.595410; this version posted May 23, 2024. The copyright holder for this preprint (which was not certified by peer review) is the author/funder, who has granted bioRxiv a license to display the preprint in perpetuity. It is made available under aCC-BY-NC-ND 4.0 International license.

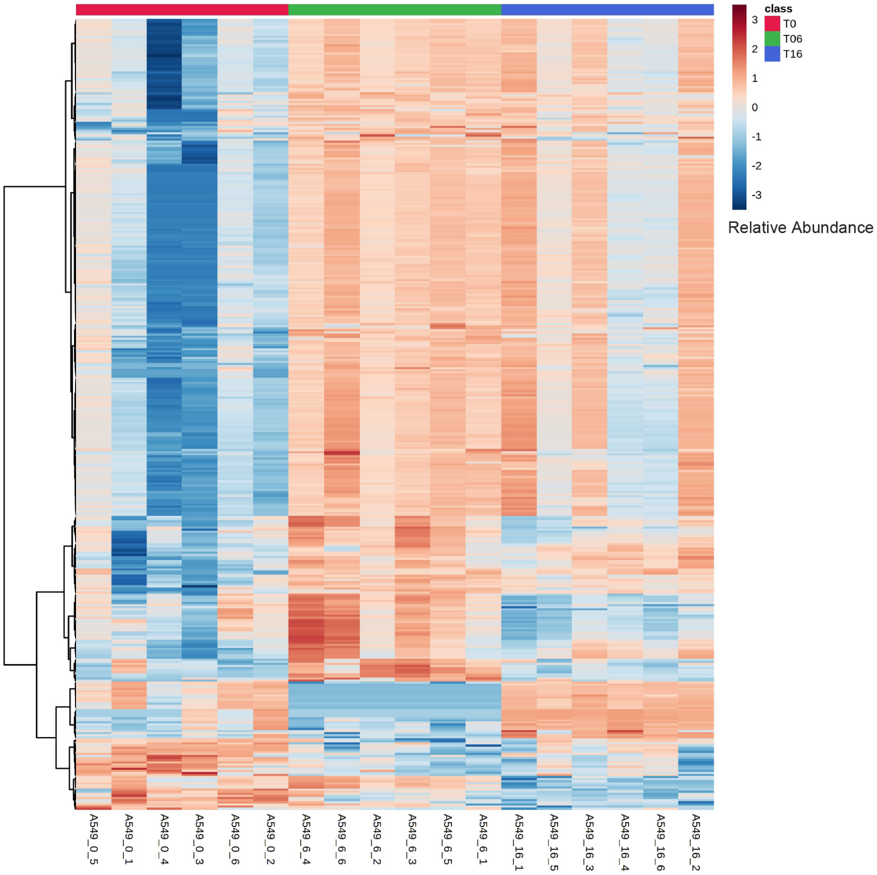

Figure S2: Heatmap analysis of significant metabolites (n = 377) for all A549 samples at each time point.
